# Cytokine profile in peripheral blood mononuclear cells differs between embryo donor and potential recipient sows

**DOI:** 10.3389/fvets.2024.1333941

**Published:** 2024-03-27

**Authors:** Josep M. Cambra, Maria A. Gil, Cristina Cuello, Alejandro Gonzalez-Plaza, Heriberto Rodriguez-Martinez, Nikolai Klymiuk, Emilio A. Martinez, Inmaculada Parrilla

**Affiliations:** ^1^Department of Medicine and Animal Surgery, Faculty of Veterinary Medicine, International Excellence Campus for Higher Education and Research “Campus Mare Nostrum”, University of Murcia, Murcia, Spain; ^2^Institute for Biomedical Research of Murcia (IMIB-Arrixaca), Campus de Ciencias de la Salud, Murcia, Spain; ^3^Large Animal Models in Cardiovascular Research, Internal Medical Department I, TU Munich, Munich, Germany; ^4^Department of Biomedical and Clinical Sciences (BKV), BKH/Obstetrics and Gynecology, Faculty of Medicine and Health Sciences, Linköping University, Linköping, Sweden

**Keywords:** embryo transfer, embryo, pig, pregnancy, PBMC, cytokines

## Abstract

**Introduction:**

Pregnancy success relies on the establishment of a delicate immune balance that requires the early activation of a series of local and systemic immune mechanisms. The changes in the immunological profile that are normally occurring in the pregnant uterus does not take place in cyclic (non-pregnant) uterus, a fact that has been widely explored in pigs at the tissue local level. Such differences would be especially important in the context of embryo transfer (ET), where a growing body of literature indicates that immunological differences at the uterine level between donors and recipients may significantly impact embryonic mortality. However, whether components of peripheral immunity also play a role in this context remains unknown. Accordingly, our hypothesis is that the immune status of donor sows differs from potential recipients, not only at the tissue local level but also at the systemic level. These differences could contribute to the high embryonic mortality rates occurring in ET programs.

**Methods:**

In this study differences in systemic immunity, based on cytokine gene expression profile in peripheral blood mononuclear cells (PBMCs), between embryo-bearing donor (DO group; *N* = 10) and potential recipient sows (RE group; *N* = 10) at Day 6 after the onset of the estrus were explored. Gene expression analysis was conducted for 6 proinflammatory (IL-1α, IL-1β, IL-2, GM-CSF, IFN-γ, and TNF-α) and 6 anti-inflammatory (IL-4, IL-6, IL-10, IL-13, TGF-β1, and LIF) cytokines.

**Results and discussion:**

All cytokines were overexpressed in the DO group except for IL-4, suggesting that stimuli derived from the insemination and/or the resultant embryos modify the systemic immune profile in DO sows compared to RE (lacking these stimuli). Our results also suggest that certain cytokines (e.g., IL-1α and IL-1β) might have a predictive value for the pregnancy status.

## 1 Introduction

From an immunological point of view, the success of pregnancy depends on a delicate paradox due to the necessary coexistence of adequate immune response to protect the mother and the embryo against harmful agents, with the establishment immunotolerance mechanisms for preventing embryo rejection and allowing correct implantation and pregnancy progress (1).

This complex balance during early pregnancy requires a series of conceptus-driven signals that ultimately trigger these immunological events (2) that are reflected locally in the endometrium but also peripherally, the latter conditioning systemic immunity through peripheral blood mononuclear cells (PBMCs) (3). The PBMCs, composed of circulating lymphocytes, monocytes and dendritic cells can react to the presence of embryos and transmit information between the endometrium and the lymphoid and endocrine organs, establishing a complex system of systemic embryo-maternal communication (4). A fundamental component of these communication mechanisms are cytokines, small proteins with immunoregulatory functions that are secreted by most cells, particularly those of the immune system (5). The expression of cytokines in PBMCs during gestational events is a very interesting field of study. The cytokines produced by T helper (Th) 2 lymphocytes such as interleukin (IL)-4, IL-5 or IL-13 have traditionally been associated with a good prognosis of pregnancy (6), given their anti-inflammatory and humoral immunity-promoting profile. In contrast, Th1 or proinflammatory cytokines such as IL-2, interferon gamma (IFN-γ) or tumor necrosis factor alpha (TNF-α) have been related to adverse pregnancy progression (7) due to stimulation of cellular immunity. However, this classical Th1/Th2 paradigm has been repeatedly questioned for decades (8, 9). Although a Th2 or anti-inflammatory response may be well established in late pregnancy, many authors agree that pro- and anti-inflammatory events may alternate throughout early pregnancy (10), revealing a much more dynamic functionality during these stages until proper implantation takes place. These immune-mediated mechanisms and the role played by the different cytokines have been extensively studied in different mammalian species (11); research that, however, has focused on the immunological effects at a local (oviduct/uterine) level, while systemic studies have been limited. In this context, investigations conducted on species with low invasive placentation, such as cattle, showed changes in the expression profile of cytokines in PBMCs in relation to the presence of embryos, both during the first days of embryonic development (12, 13) and later around implantation (14, 15). In pigs, it is known that the presence of uterine day-6 blastocysts promotes immune tolerance for embryos (16–18), but their impact at systemic level remains unknown. Increasing our understanding of these mechanisms is particularly important for porcine embryo transfer (ET) programs as immunological differences at uterine level between donors and recipients can have a significant impact on reproductive outcomes (18–20). We hypothesize that these differences are also present at the immune systemic level, which could further contribute to the high embryonic mortality following ET (21).

The present study aimed to demonstrate that Day 6 immune systemic response in donor sows (artificial inseminated and embryo bearing sows) is different from that of potential recipient sows (uninseminated, no embryo presence). This difference, due most probably to the inseminate (by artificial insemination (AI) components) and/or to the presence of embryos, would trigger an immune systemic response that could be detected by gene expression changes of specific cytokines in circulating PBMCs. Furthermore, changes in PBMC-cytokine expression could potentially serve as early indicators of pregnancy status. Additionally, increased knowledge of these immunological aspects could relevantly help to improve ET outputs in swine since it could make possible to condition or immunologically prepare the recipient sows for a better acceptance of the transferred embryos.

## 2 Materials and methods

Unless otherwise indicated, all reagents and chemicals used during this study were supplied by Sigma–Aldrich Co. (Alcobendas, Madrid, Spain). The animal study protocol was approved by the Ethics Committee for experiments with animals of the University of Murcia (Code: 850/2023).

### 2.1 Animal management and housing

Peripheral blood samples were retrieved for PBMC isolation from weaned multiparous Landrace x Large-White sows (4–6 parities) with similar lactation periods (21–24 days). The sows were housed in individual pens at a local pig farm (Agropor SL, Murcia, Spain) under controlled temperature and humidity, with *ad libitum* access to a water source and with being fed twice a day according to their nutritional requirements. All sows had body condition scores ranging from 2.75 to 3.25 on a five-point scale on the day of weaning. Sexually mature Duroc boars from an insemination center (AIM Iberica, Murcia, Spain) were used to obtain the seminal doses to perform AI.

### 2.2 Reproductive management: estrus detection and AI

Estrus detection started 1 day after weaning and was performed daily by a skilled operator using a vasectomized male to detect behavioral changes in the females. Those females that presented a standing reflex were considered in estrus, and this day was considered Day 0. Because the experiment was carried out in the context of an ET program, all sows were superovulated with 1,000 IU eCG (i.m.; Folligon, Intervet International B.V., Boxxmeer, Netherlands) 24 h after weaning followed by 750 IU hCG (i.m.; VeterinCorion, Divasa Farmavic, S.A., Barcelona, Spain) 72 to 96 h later at the onset of estrus. A total of 28 multiparous sows were weaned on the same day. Only sows with a weaning-to-estrus interval of 4–5 days (*N* = 21) were used for the experiment. For the donor sows’ group (DO group), 11 randomly selected sows were inseminated following estrus detection, while the remaining 10 sows were left without insemination and constituted the group of potential recipient sows (RE group). Insemination was performed cervically 6 and 24 h after the onset of estrus using AI doses (90 mL) containing 3 × 10^9^ spermatozoa.

### 2.3 Blood collection and PBMC isolation

Blood samples from all sows were collected 6 days after the onset of estrus, at the same time of the day and always before surgery in the case of DO sows. Samples were collected in EDTA-containing Vacutainers by jugular vein puncture. PBMC isolation was performed by density-gradient centrifugation as previously reported (22). First, 5 mL of blood was expanded (1:1) with PBS, carefully layered over 3 mL of Histopaque solution (density 1.077 g/mL) and centrifuged at 900 × g for 30 min without brakes. After centrifugation, the layer between the serum and the Histopaque solution (buffy coat) was collected and transferred to a clean tube containing 10 mL of PBS. After further centrifugation (350 × g for 10 min), the supernatant was removed, and another 10 mL of fresh PBS was added to complete the washing process. The resulting pellet was resuspended in 2 mL of RPMI medium supplemented with nonessential amino acids, glutamine, antibiotics and 10% fetal calf serum. A total of 2 × 10^6^ isolated PBMCs from each sow were preserved in RNAlater at −80°C until use.

### 2.4 RNA isolation and gene expression analyses

Isolation of RNA was performed using the RNeasy Plus Micro Kit (Qiagen, Hilden, Germany) following the manufacturer’s instructions. The quantity and quality of the isolated RNA was tested with a NanoDrop spectrophotometer (Thermo Fisher Scientific, Madrid, Spain). The Maxima H Minus First Strand cDNA Synthesis Kit (Thermo Fisher Scientific, Waltham, Massachusetts, United States) was used for complementary DNA synthesis using a thermocycling protocol consisting of an initial step at 25°C for 10 min, followed by retro-transcription at 50°C for 15 min and concluding with inactivation at 85°C for 5 min in an Eppendorf Mastercycler (Eppendorf AG, Hamburg, Germany). Primer3Plus software (23) was used to design the primers for the different genes studied. The sequences of these primers are shown in Table 1. The Ensembl (24) and NCBI Resource Coordinators (25) databases were used to obtain sequence information, exon status and the different isoforms described for each gene. For primer design, it was considered that the position of the primers should be in cross exon–exon boundaries or in different exons to avoid nonspecific amplifications of genomic DNA, and in the case of genes with several isoforms, the primers were located in exons common to all isoforms to avoid differences attributable to differential isoform expression. Prior to gene expression analyses, the efficiency of each primer pair was evaluated by calculating the slope of the regression line of the Ct values obtained from a 5-serial sample dilution. The slope value obtained was transformed to obtain the amplification factor for each reaction, ranging from 1.9 to 2.1. The specificity of the primers was tested by running an agarose gel and checking that the size of the PCR products matched the theoretical size of the amplicons (Supplementary Figure S1). iTaq Universal SYBR Green Supermix (Applied Biosystems, Foster City, CA, United States) was used to perform qPCR. The final reaction mixture contained 5 μL of 2X Green Supermix, 2 μL of cDNA, 1 μL of each primer pair (500 nM) and 1 μL of dH_2_O. The reactions were carried out in duplicate in a QuantStudio 5 Real-Time PCR System (Applied Biosystems, Foster City, CA, United States) with a thermocycling profile as follows: initial UDG activation at 50°C (2 min) and a previous denaturation step at 95°C (2 min). These steps were followed by 40 cycles of denaturation at 95°C (5 s) and annealing at 60°C (30 s). A final melt curve analysis was performed to confirm the PCR specificity by the detection of a single distinct peak. The normalized gene expression of each target gene was calculated based on the method described by Ganger et al. (26), using the expression of the genes beta-actin (ACTB), glyceraldehyde-3-phosphate dehydrogenase (GAPDH) and peptidylprolyl isomerase A (PPIA) as endogenous controls.

**Table 1:** Primer sequences for the analysis of mRNA gene expression.

Gen	Accession number	Primers (5′– 3′)	Size	Amplification factor
IL-1α^1^	NM_214029	F: ATGCCCGCAATCAAAGCATC	195	2.03
		R: ACACGGGTTCGTCTTCGTTT		
IL-1β^1^	NM_001302388.2	F: CTTAGGGATCAAGGGAAAGAA	202	1.94
		R: TTGAGAGGTGCTGATGTACCA		
IL-2^1^	NM_213861.1	F: TACATGCCCAAGCAGGCTA	169	1.97
		R: CAGATCCCTTTAGTTCCAAAACTG		
GM-CSF^1^	NM_214118.2	F: CAGACTCGCCTGAACCTGTA	198	2.08
		R: GCAGTCAAAGGGGATGGTAA		
IFN-γ^1^	NM_213948.1	F: TCAAAGGAGCATGGATGTGA	203	2.03
		R: TCTGACTTCTCTTCCGCTTTCT		
TNF-α^1^	EU682384.1	F: CGTTGTAGCCAATGTCAAAGC	194	2.07
		R: TGGTGTGAGTGAGGAAAACG		
IL-4^2^	NM_214123.1	F: GTGCGACATCACCTTACAAGAG	70	1.95
		R: CATGCACGAGTTCTTTCTCG		
IL-6^2^	NM_214399.1	F: CTCGGCAAAATCTCTGCAAT	168	2.10
		R: GGTGATTCTCATCAAGCAGGT		
IL-10^2^	NM_214041.1	F: TGGAAGACGTAATGCCGAAG	141	1.97
		R: CCTTGCTCTTGTTTTCACAGG		
IL-13^2^	NM_213803.1	F: TCCAAAAGACCCAGAGGATG	155	2.03
		R: CTACCCGTGGCGAAAAATC		
TGF-β1^2^	NM_214015.2	F: GGACTACTACGCCAAGGAGGT	159	2.00
		R: TCTGCCCGAGAGAGCAATAC		
LIF^2^	AY585336.1	F: GCCAACGCCCTCTTTATTCT	222	2.01
		R: GTTCACAGCACCAGGATTGA		
ACTB^3^	XM_003357928.4	F: CTCGATCATGAAGTGCGACGT	114	1.96
		R: GTGATCTCCTTCTGCATCCTGTC		
GAPDH^3^	NM_001206359.1	F: ATCACTGCCACCCAGAAGAC	194	1.97
		R: AGATCCACAACCGACACGTT		
PPIA^3^	XM_021078519.1	F: CTGAAGCATACGGGTCCTGG	100	2.02
		R: CCAACCACTCAGTCTTGGCA		

^1^Proinflammatory cytokines. ^2^Anti-inflammatory cytokines. ^3^Endogenous controls.

### 2.5 Surgical embryo collection

To confirm the presence of embryos in the inseminated sows, surgical embryo collections were performed 6 days after the first insemination. Embryo collection was performed as previously described by Martinez et al. (27). Briefly, inseminated sows were sedated by intramuscular administration of azaperone (2 mg/kg body weight). Anaesthesia induction was done by intravenous administration of sodium thiopental (7 mg/kg body weight) and maintained by inhalatory administration of isoflurane (3–5%). By means of a laparotomy, the genital tract of the sows was exposed, accessing the proximal portion of the uterine horns for flushing with 30 mL of saline medium. After collection, the embryos were washed and classified in terms of stage and quality according to the criteria of the International Embryo Transfer Society (28). Due to the day of collection, only those sows with embryos at the morula or blastocyst stage were considered for the experiment. Of the 11 sows inseminated, 10 met these criteria, so they were finally used to form the DO group.

### 2.6 Validation and quality assurance

Concentration and viability of the PBMCs obtained were calculated by Trypan Blue staining, yielding an average concentration of 10 × 10^6^ cells/mL, of which over 95% were viable. The purity of the isolated PBMCs was confirmed by flow cytometry (BD FACS Canto II flow cytometer; Becton Dickinson & Company). Based on cell morphology using dot-plot representing Forward Scatter Area (FSC-A) vs. Side Scatter Area (SSC-A), blood samples from the same animal were analyzed with and without the PBMC isolation protocol, showing in the first case, the segregation between lymphocytes and monocytes and the absence of granulocytes, thus validating the isolation protocol. The quantity and quality of the isolated RNA was validated with a NanoDrop spectrophotometer (Thermo Fisher Scientific, Madrid, Spain) accepting those samples whose 260/280 and 260/230 ratios were between 1.8 and 2.2. The efficiency of each primer pair was evaluated by calculating the regression line for the Cq values of 5 serial dilutions for each primer pair reaction. The slope values obtained were transformed to obtain the amplification factor for each reaction, with values comprised between 1.9 and 2.1 with R2 values greater than 0.99. The specificity of the primers was tested by running an agarose gel. PCR products (2 μL) were mixed with loading dye (2 μL) and H2O (8 μL) and loaded in a 2% agarose gel with SYBR Safe DNA Gel Stain (Thermo Fisher Scientific, Madrid, Spain). Samples were separated by electrophoresis for 50 min at 120 V in Tris acetate EDTA buffer. An UV transilluminator was used to visualize the gel bands checking that the size of the PCR products matched the theoretical size of the expected amplicons (Supplementary Figure S1). The suitability of the endogenous genes used for gene expression calculations was validated by comparing the cDNA amount of each sample with the Cq value obtained for the 3 genes. A linear regression analysis determined *R*^2^-values of 0.76, 0.88, and 0.91 for ACTB, GAPDH and PPIA, respectively (Supplementary Figure S2). Despite the better stability observed for the endogenous PPIA gene, the analyses showed no difference between single normalization with PPIA or with the mean of the three genes, so we finally opted for this second approach to avoid the probability of bias due to the use of a single endogenous gene.

### 2.7 Statistical analysis of results

The lognormality of the expression values for each of the genes studied was analyzed using a Shapiro–Wilk test, showing a parametric distribution for each of the variables. Statistical significance between the two experimental groups was determined by pairwise comparison with a Student’s *t*-test. The *p*-value obtained was adjusted using the Benjamini–Hochberg procedure (False Discovery Rate). The value of the AUC and ROC curves in the DO and RE groups were estimated for different cytokine gene expression values using the Wilson/Brown method. The correlations between gene expression and the number of prehatching embryos were compared using Pearson’s correlation. Differences were considered significant at *p* < 0.05. The statistical analyses were conducted using GraphPad PRISM version 8.0 (GraphPad Software, Inc., San Diego, CA). Principal component and hierarchical clustering analyses were calculated using the web tool ClustVis (29).

## 3 Results

### 3.1 Reproductive parameters

The number of corpora lutea in the DO sows ranged from 30 to 44 with a mean ovulation rate of 36.8 ± 4.3. A total of 324 embryos (range, 26–41) and 19 oocytes/degenerated embryos (range, 0–5) were recovered (recovery rate, 93.2%). Of the 324 embryos collected, 67.6 and 32.4% were morulae and unhatched blastocysts, respectively. Individualized information for each of the animals in the DO group can be found in Table 2.

**Table 2:** Reproductive parameters obtained in the DO group.

Donor animal	Corpora lutea	Cysts	Morulae	Blastocysts	Oocytes/degenerated embryos
DO1	30	0	0	35	0
DO2	38	0	0	35	5
DO3	37	0	35	0	0
DO4	32	0	26	0	0
DO5	36	0	29	0	0
DO6	33	1	30	0	2
DO7	37	0	26	0	0
DO8	44	0	37	0	3
DO9	42	0	40	0	2
DO10	39	0	30	0	8

### 3.2 Sample profiling based on the multivariate analyses

**Figure 1:**
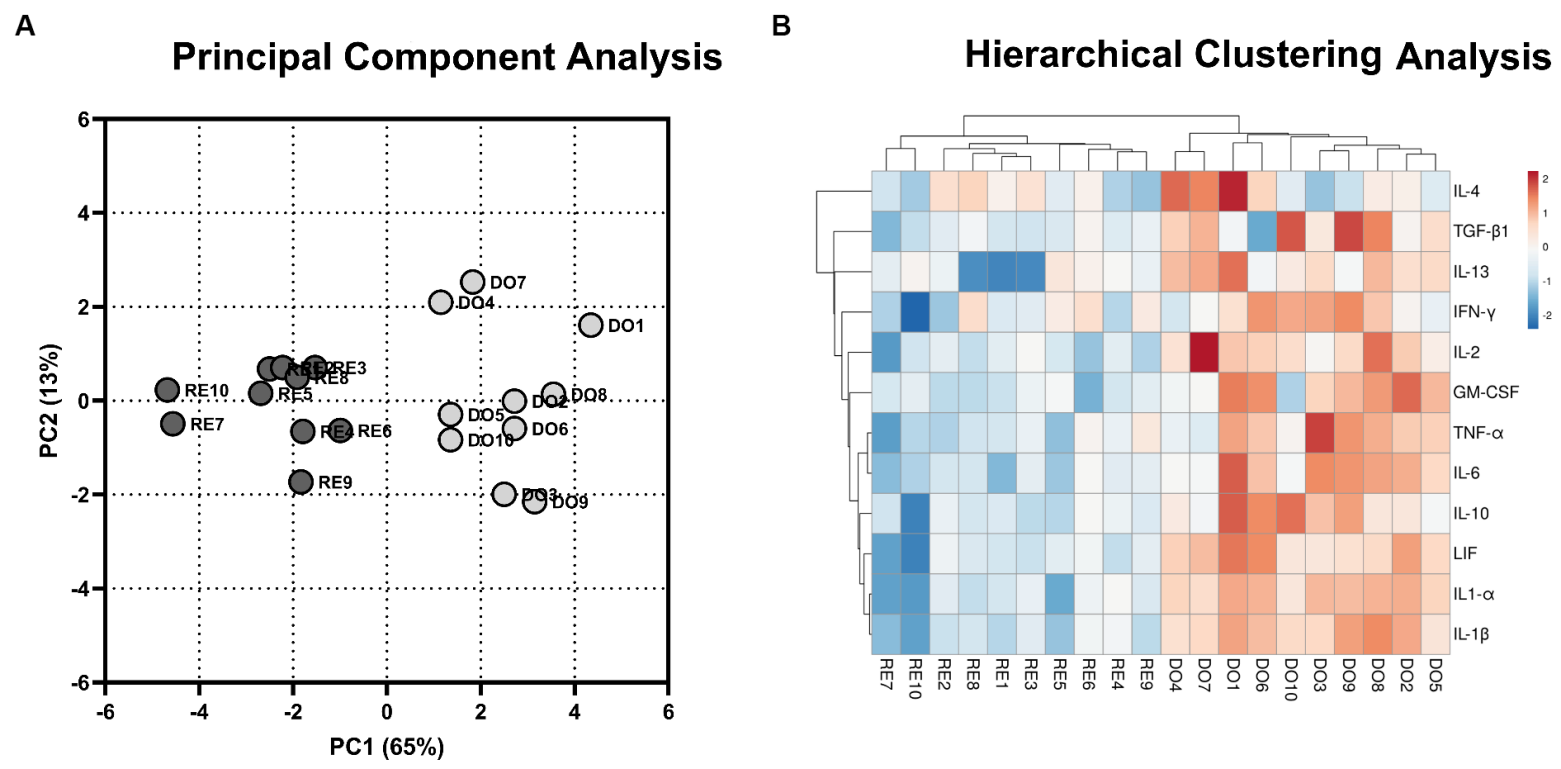
Multivariate analyses. **(A)** 2D plot representation of the principal component analyses. The gray dots represent donor sows (DO; *N* = 10) samples, whereas the black dots represent the samples of the potential recipient sows group (RE; *N* = 10). The first 2 principal components (PC) explain 78% of the variability of the data (PC1, 65%; PC2, 13%). **(B)** Hierarchical clustering analysis comparing the gene expression of the PBMCs for DO- and RE- derived samples. Rows and columns represent genes and samples, respectively. The color intensity corresponds to the expressions of the cytokines studied; warm colors indicate higher expression and cool colors indicate lower expression. The upper branches indicate the relationships among the samples, and the left branches indicate differences among the analyzed genes.

To analyze the distribution of the DO and RE samples, a principal component analysis was performed with the gene expression data of the 12 cytokines studied. This analysis grouped the samples into two distinct population clusters, corresponding to their respective experimental groups (Figure 1A). The results of the analysis revealed that the first two components explained 78% of the variation. Using hierarchical clustering analysis, the expression of the genes studied showed a similar segregation between the samples corresponding to the DO and RE groups. Most DO samples had higher gene expression, indicating a more active profile (Figure 1B).

### 3.3 Genic expression of proinflammatory cytokines

**Figure 2:**
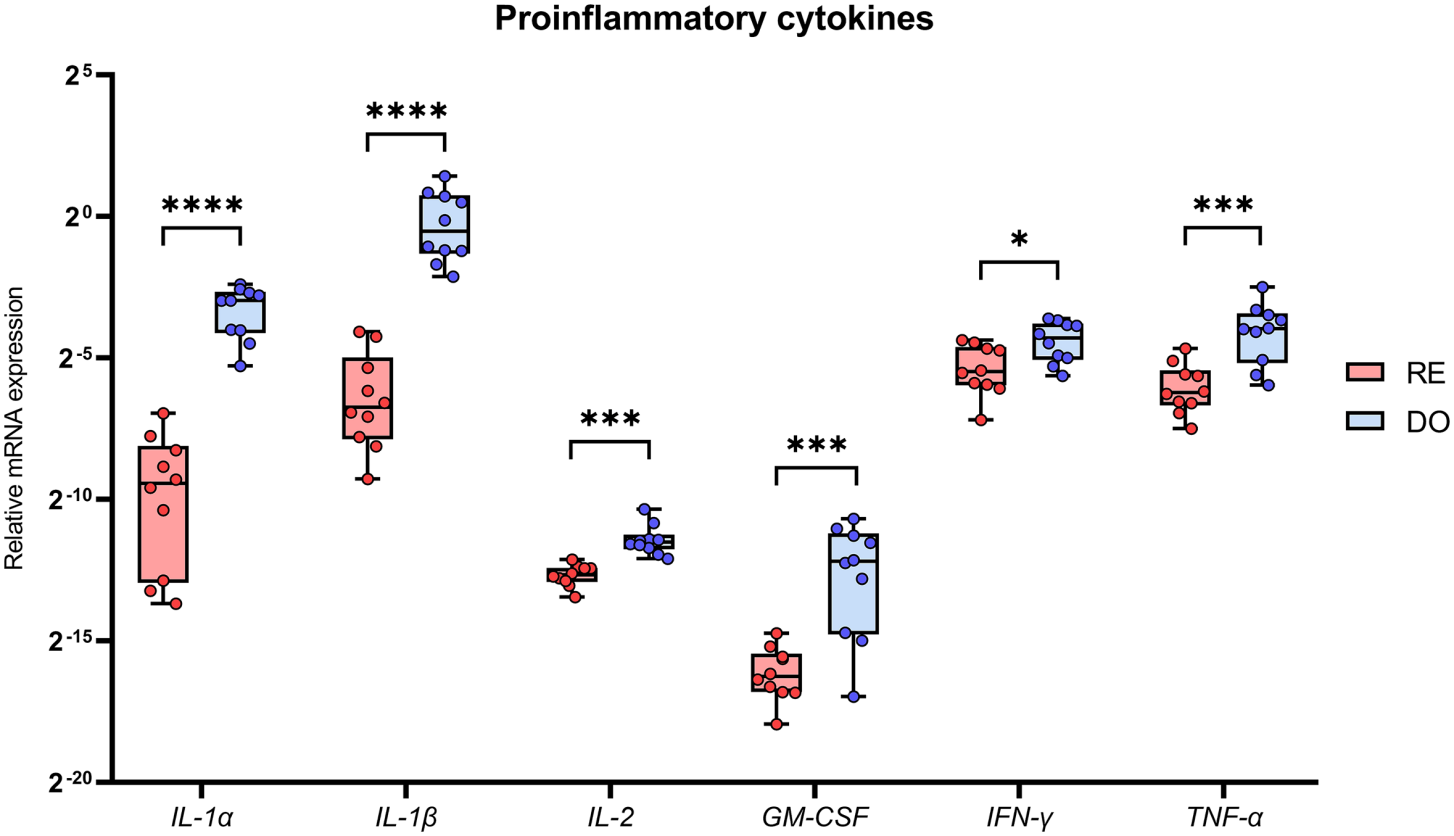
Relative mRNA expression in porcine PBMCs of the proinflammatory cytokines. Interleukin 1 alpha (IL-1α), interleukin 1 beta (IL-1β), interleukin 2 (IL-2), granulocyte macrophage colony stimulating factor (GM-CSF), interferon gamma (IFN-γ) and tumor necrosis factor alpha (TNF-α). The superscripts indicate significant differences (*p < 0.05, ***p < 0.001, and ****p < 0.000001) between embryo-bearing (DO) and nonpregnant (RE) control sows at Day 6 of the cycle. Data are represented as the median ± interquartile range.

PBMCs isolated from the DO group showed significantly higher expression for all analyzed cytokines classified with a proinflammatory profile compared with RE sows (Figure 2). Noteworthy were the comparisons made with the cytokines belonging to the IL 1 superfamily, IL-1α and IL-1β, which showed the highest expression levels in the DO group as well as the most significant differences (*p* < 0.000001) compared with the RE group.

### 3.4 Genic expression of anti-inflammatory cytokines

**Figure 3:**
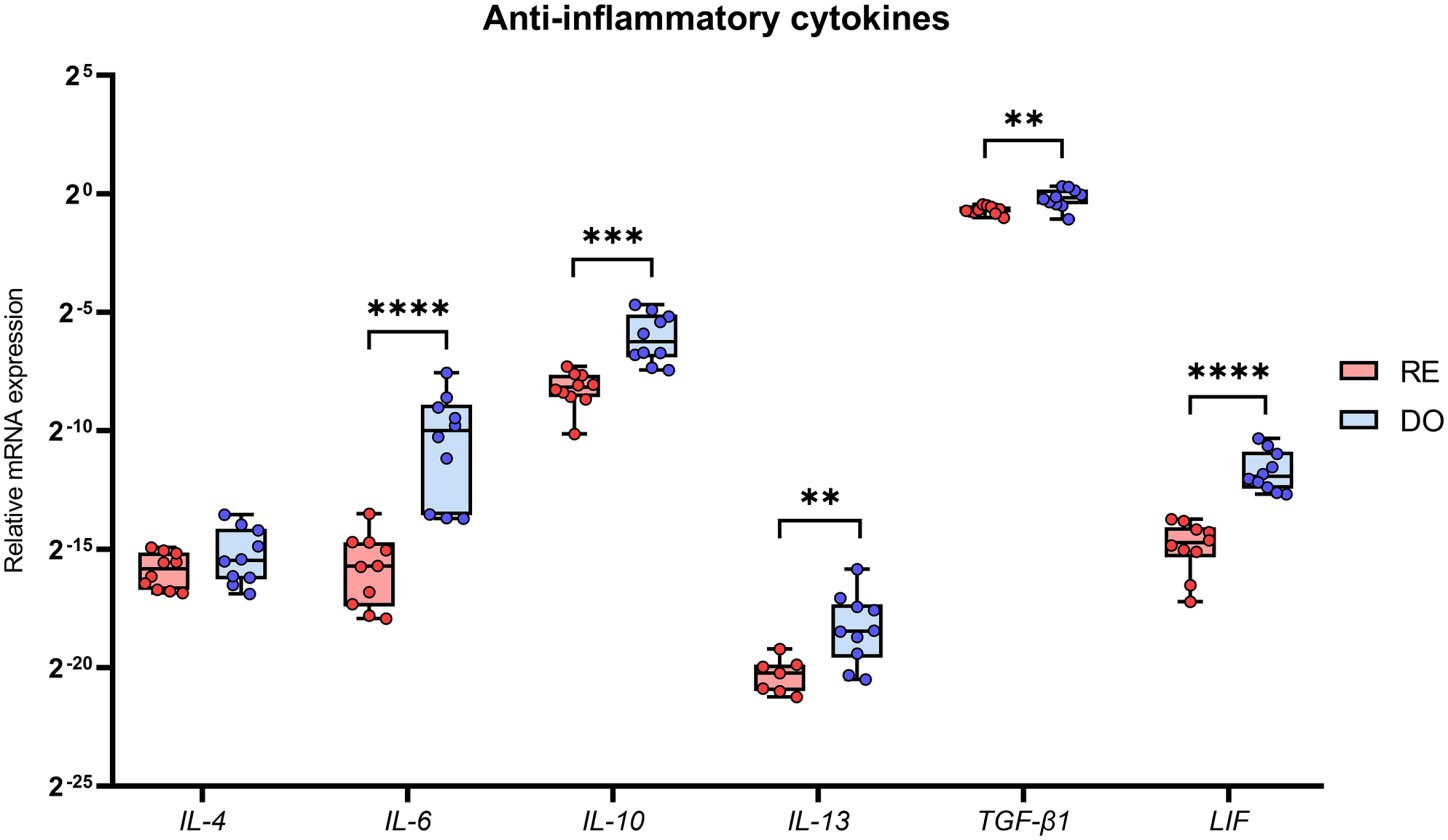
Relative mRNA expression in porcine PBMCs of the anti-inflammatory cytokines. Interleukin 4 (IL-4), interleukin 6 (IL-6), interleukin 10 (IL-10), interleukin 13 (IL-13), transforming growth factor beta 1 (TGF-β1), and leukemia inhibitory factor (LIF). The presence of superscripts indicates significant differences (**p < 0.01, ***p < 0.001, and ****p < 0.0001) between embryo-bearing (DO) and nonpregnant (RE) control sows at Day 6 of the cycle. Data are represented as the median ± interquartile range. Note that there are 3 missing values in the case of IL-13 group RE (*N* = 7), as the expression values obtained were too low for comparison.

All cytokines with an anti-inflammatory profile, except for IL-4, showed significantly higher expression in the PBMCs of the DO sows than those in the RE sows (Figure 3). The cytokines showing the greatest differences between groups were IL-6 and LIF, with *p*-values below 0.0001.

### 3.5 Receiver operator characteristic curves and area under curve analyses

**Figure 4:**
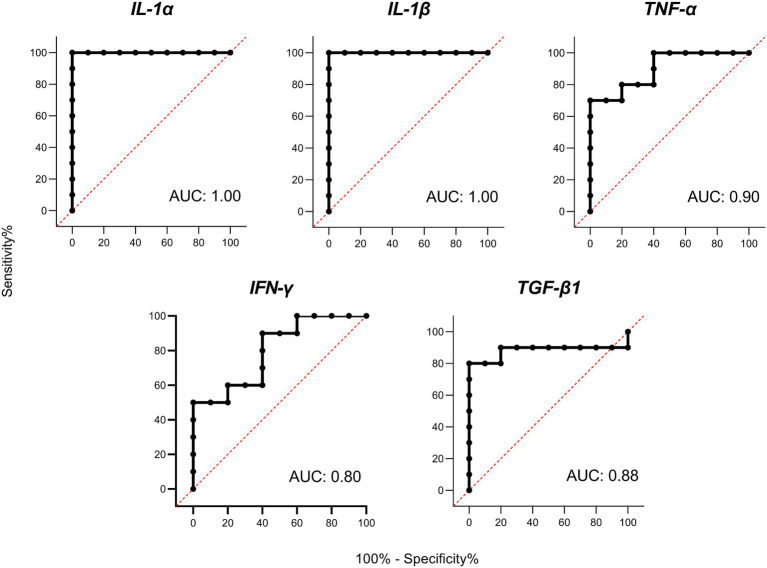
Receiver operator characteristic (ROC) curves and area under curve (AUC) analyses. ROC curves for porcine interleukin 1 alpha (IL-1α), interleukin 1 beta (IL-1β), tumor necrosis factor alpha (TNF-α), interferon gamma (IFN-γ), and transforming growth factor beta 1 (TGF-β1) based on PBMC gene expression on Day 6 of the cycle.

Receiver operator characteristic (ROC) curves were calculated for the 5 differentially expressed cytokines with the highest levels of gene expression (IL-1α, IL-1β, TNF-α, IFN-γ, and TGF-β1; Figure 4). The ROC curves for the five cytokines analyzed reached high levels of significance (*p* < 0.005), demonstrating that an adequate cut-off value can be established. It should also be noted that the cytokines belonging to the IL-1 family presented the highest AUC values, suggesting a very high predictive value under the conditions studied.

### 3.6 Correlation between gene expression and the number of collected embryos in the do sows

**Figure 5:**
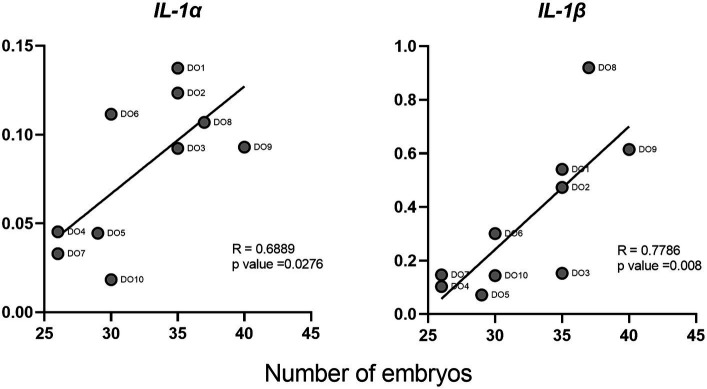
Correlations between interleukin 1 alpha (IL-1α) and interleukin 1 beta (IL-1β) cytokines and the number of embryos in donor sows. Each point represents the different DO samples analyzed (*N* = 10).

We evaluated the correlations between the normalized gene expression levels in the PBMCs of the DO group and the number of embryos collected from each of the sows. Two cytokines (IL-1α and IL-1β) showed a significant positive correlation between their expression and the number of embryos (Figure 5). IL-1β showed the strongest correlation with an R value of 0.78.

## 4 Discussion

To the best of our knowledge, this study is the first to describe cytokine gene expression in PBMCs in the context of a pig ET program. The results demonstrate large differences in the expression profiles between embryo bearing donor sows and potential recipient sows as early as Day 6 of the cycle, the commonly accepted day for the transfer of morula-blastocyst stage embryos in this species (30). Multivariate analyses confirm that the expression pattern of the analyzed cytokines clearly segregates the PBMCs between sow groups, showing a much more active profile of these cells in the DO sows. All proinflammatory and anti-inflammatory (except for IL-4) cytokines were overexpressed by the PBMCs in the DO sows, which largely agrees with a previous study showing that the porcine endometrium increases cytokine expression at Day 6 of the cycle in embryo bearing sows (18).

In an ET context the main differences between DO and RE sows are the subsequent presence of the insemination components and/or of the resulting embryos in their genital tracts; either of which could (or could in combination) be responsible for the differential PBMC cytokine gene expression. In pigs, it is widely accepted that AI-dose components can alter the transcriptome of the endometrium and embryos, affecting important genes related to immune processes, at least up to Day 6 after insemination (31, 32). However, whether these signals generate an impact at the systemic level is a concept that has barely been explored, being only one report in pigs (17) which suggests that seminal plasma infusions during estrus do not exert any significant change in circulating lymphocyte populations in sows at Day 6 of the cycle. On the other hand, there is an extensive literature on the embryonic influence on the conditioning of the uterine environment and how this response could be transferred to the systemic level. Supporting this suggestion, many reports analyze the numerous factors secreted by the embryo, and unlike the seminal components present only in the first days, these would be secreted in a continuous and more prolonged manner, generating greater influence on the uterine environment. For instance, during the preblastocyst stages, human embryos secrete cytokines (IL-1, IL-6, and LIF) and other molecules such as human leukocyte antigen G or human chorionic gonadotropin (33). In relation to this last factor, different studies indicate how this factor is capable of modifying the expression of cytokines in PBMCs, thus proposing human chorionic gonadotropin as a possible factor in the systemic effects during early gestation in humans [reviewed by Ott and Gilfford (3)]. This mechanism seems to be clearer in ruminants, where the key embryonic factor would be IFN-τ. Transcripts of this factor can be secreted by bovine 16-cell stage embryos (34) and there is also evidence that IFN-τ from embryonic origin is able to enter into the blood via uterine vessels and then modify the gene expression of peripheral blood immune cells (35). In relation to swine species, early pig embryos (Days 2–6) can influence the transcriptomic (16, 36) and proteomic (37) profiles of the oviductal/uterine tract, affecting immune-related genes. However, there are no reports about how these local immune changes influence the peripheral immune response. Both endocrine and proinflammatory immunological mediators such as estrogens, IL-1β and IL-6 are secreted by the pig embryos before the implantation (38). While estrogens (39) and IL-6 (40) have been detected during later stages, Mathew et al. (38) identified IL-1β transcripts in day 6 swine blastocysts. In particular, this cytokine would play a fundamental role in early embryonic development in pigs, being necessary for proper embryonic elongation and influencing the subsequent maternal recognition of pregnancy (41). Hence, IL-1β could be equivalent to IFN-τ in ruminants, and in the case of swine it could be one of the embryonic factors related to immune modulation at the systemic level. Therefore, although we cannot completely exclude a systemic effect of the AI dose, it seems that the embryonic presence and particularly specific embryo secreted factors, could be the main responsible of the encountered differences in the gene expression profiles of PBMCs between DO and RE sows at this time point of the cycle.

Cytokine expression in PBMC has been already analyzed in previous studies in pregnant and non-pregnant cows (12, 42) on days 7–8 of the cycle, similarly to our DO and RE groups, respectively. Like our findings, they found that IL-10 and TGF-β1 expressions were increased in PBMCs at pregnancy. In the context of maternal recognition in pigs, both anti-inflammatory cytokines develop an important immunosuppressive effect and also have been linked with pregnancy impairment under altered levels (43, 44). It is also generally accepted that they have a critical role for inducing the naïve CD4+ T, in order to differentiate them into T regulatory cells (45, 46), basic to induce an immunotolerant response (47). The overexpression of both cytokines in DO sows could be related with the requirement to potentiate this lymphocyte subcluster during this time, which will be critical to recognize the embryonic alloantigens during the implantation process, thus avoiding an immune response against them. A reduced number of these specific lymphocytes in the potential recipient sows due to low expression of these cytokines could explain the high embryonic lethality, since the immunotolerant phenomena necessary for implantation would be impaired at this critical moment. On the other side the increased expression of pro-inflammatory cytokines in PBMCs of DO differs to what was observed in cows (12, 42). For instance, the TNF-α expression did not show significant differences between embryo-bearing and nonpregnant cows, while IL-1β was downregulated in the former group. These discrepancies may result from differences in the cytokine dynamics between the two species. For example, as we already mention, IL-1β is secreted by the swine embryos and is requested for its proper development, while in ruminants its role seems to be minor at the embryonic level. Another difference between these studies was the number of embryos present in the uterine lumen. In our study, the average number of embryos collected per superovulated sow was above 30, which is three times the number of embryos obtained in superovulated cows (12, 13). Therefore, we can speculate that the number of embryos could also explain the different cytokine expression between species. Overall, with the results obtained in swine, it seems that immunomodulation during preimplantation stages in embryo bearing sows could be a dynamic process, where both Th1 and Th2 response coexists in a balanced equilibrium, which aligns perfectly with an increasing number of studies supporting the idea that successful pregnancy requires highly dynamic immune response rather than a suppressive one (48). Additionally, the particular increase in pro-inflammatory cytokine expression in DO, which seems not to match with the results in bovine, could be partially explained by the hypothesis proposed by Ott and Gifford (3) on the possibility that conceptus signals may play a role counteracting the immunosuppressive effects of progesterone. Thus, while progesterone-mediated uterine immunosuppression would be necessary for the endometrial receptivity, different compensatory measures should be activated for protecting the uterus against external agents that could jeopardize the success of the pregnancy. In line with this approach, McLendon et al. (49), describe how particularly in swine, a controlled inflammation at the endometrial level is necessary for the correct remodeling of the endometrium, as a previous step for the correct embryo implantation. In particular, this process would be mediated by the embryonic secretion of the Th1 cytokine INF-γ, which would actively recruit locally different subpopulations of T and NK lymphocytes, thus breaking with the classic paradigm that an immunosuppressive state is necessary for gestation. In any case, none of these mechanisms would take place in RE sows, resulting in a uterine environment unsuitable to accept allogeneic embryos during ET. This fact could be partially responsible for the increased embryo losses before implantation associated with ET in swine (21). Further studies are necessary to recreate the physiological immune environment at tissue local and systemic levels in the potential recipient females as a tool for improving the outputs of ET programs.

On the other hand, the role that cytokines may play as biomarkers of early pregnancy should be highlighted. Several studies have correlated the levels of IL-1β and TNF-α in endometria prior to ET with clinical pregnancies in humans (50, 51). In cattle, the expression of IFN-τ-stimulated genes (ISGs) in blood leukocytes has been used as a method of pregnancy detection, proving that genes such as Mx2 and Oas1 can be used for the early detection of pregnancy (Day 18), in heifers (52). Similarly, a recent study (53), proved the usefulness of this markers in whole blood as a reliable tool to predict the ET failure in cattle at day 21. Finally, also in cattle but at much earlier stages of gestation, the predictive value of both ISGs and IL-10 in bovine PBMCs 8 days after insemination has been suggested (12). Our results indicate that PMBC expression of some cytokines such as IL-1α, IL-1β, TNF-α, IFN-γ, and TGF-β1 might be promising candidates as markers for the presence of unhatched embryos in swine as early as 6 days after insemination, owing to the significant differences found in the gene expression of these cytokines between DO and RE sows and because of their high levels of expression. We focused on IL-1β, due to its important role during first step of pregnancy in swine species (41). Additionally, according to our results, IL-1β is the cytokine with the highest gene expression levels and also shows the highest correlation with the number of embryos. Correlation coefficient value for this cytokine and the number of embryos was 0.78, which in the context of experimental studies is considered as indicator of a high correlation between the variables studied (54). With these results at the transcriptomic level, we can infer that IL-1β gene expression has considerable potential as an indicator of the number of embryos in pregnant sows. However, transferring these studies to the serum protein level, with a larger number of animals, and also discarding some other sources of inflammation is a fundamental requirement to confirm its validity. Finding reliable markers that allow the early diagnosis of pregnancy and therefore contribute to the early detection of AI or sow fertility failures would improve the productive and reproductive indexes of swine farms. The possibility of estimating the number of embryos could also be useful not only for on-farm decisions but also for ET programs where only those sows bearing a higher number of embryos would be designed as donors, thus helping to optimize embryo collection process.

## 5 Conclusion

In conclusion, the cytokine gene expression of sow PBMCs on Day 6 of the cycle greatly differs between DO and RE sows. This difference is characterized by an overexpression of both pro- and anti-inflammatory cytokines in donors, which reflects intense changes in the immune dynamics at these very early pregnancy stages. The difference in the expression of these cytokines between DO and RE sows has important implications, since could be related to the low success rates associated with ET in this species. A better understanding of these immune mechanisms could be the key to designing strategies, such as the peripheral immune modulation, to improve uterine receptivity and pregnancy establishment for potential recipient sows. On the other hand, the results suggest a potential role of some cytokines for their use as biomarkers for the early detection of pregnancy and the number of embryos present in the porcine uterus. Further studies are needed to elucidate the mechanisms that trigger these variations in peripheral immunity as well as to validate the use of specific cytokines as pregnancy biomarkers.

## Data availability statement

The raw data supporting the conclusions of this article will be made available by the authors, without undue reservation.

## Ethics statement

The animal studies were approved by Ethics Committee for experiments with animals of the University of Murcia (Code: 486/2018). The studies were conducted in accordance with the local legislation and institutional requirements. Written informed consent was obtained from the owners for the participation of their animals in this study.

## Author contributions

JC: Conceptualization, Methodology, Writing – original draft. MG: Methodology, Writing – review & editing. CC: Methodology, Writing – review & editing. AG-P: Methodology, Writing – review & editing. HR-M: Funding acquisition, Writing – review & editing. NK: Writing – review & editing. EM: Conceptualization, Funding acquisition, Methodology, Writing – review & editing. IP: Conceptualization, Methodology, Project administration, Supervision, Writing – review & editing.
