# Supplementary material for: Cytokine profile in peripheral blood mononuclear cells differs between embryo donor and potential recipient sows
Source: Front Vet Sci. 2024 Mar 27;11:1333941. doi: 10.3389/fvets.2024.1333941 (PMC11006195; doi:10.3389/fvets.2024.1333941)
Supplement: Supplementary file 1 [file Data_Sheet_1.DOCX]

Supplementary Material

## Supplementary Figures


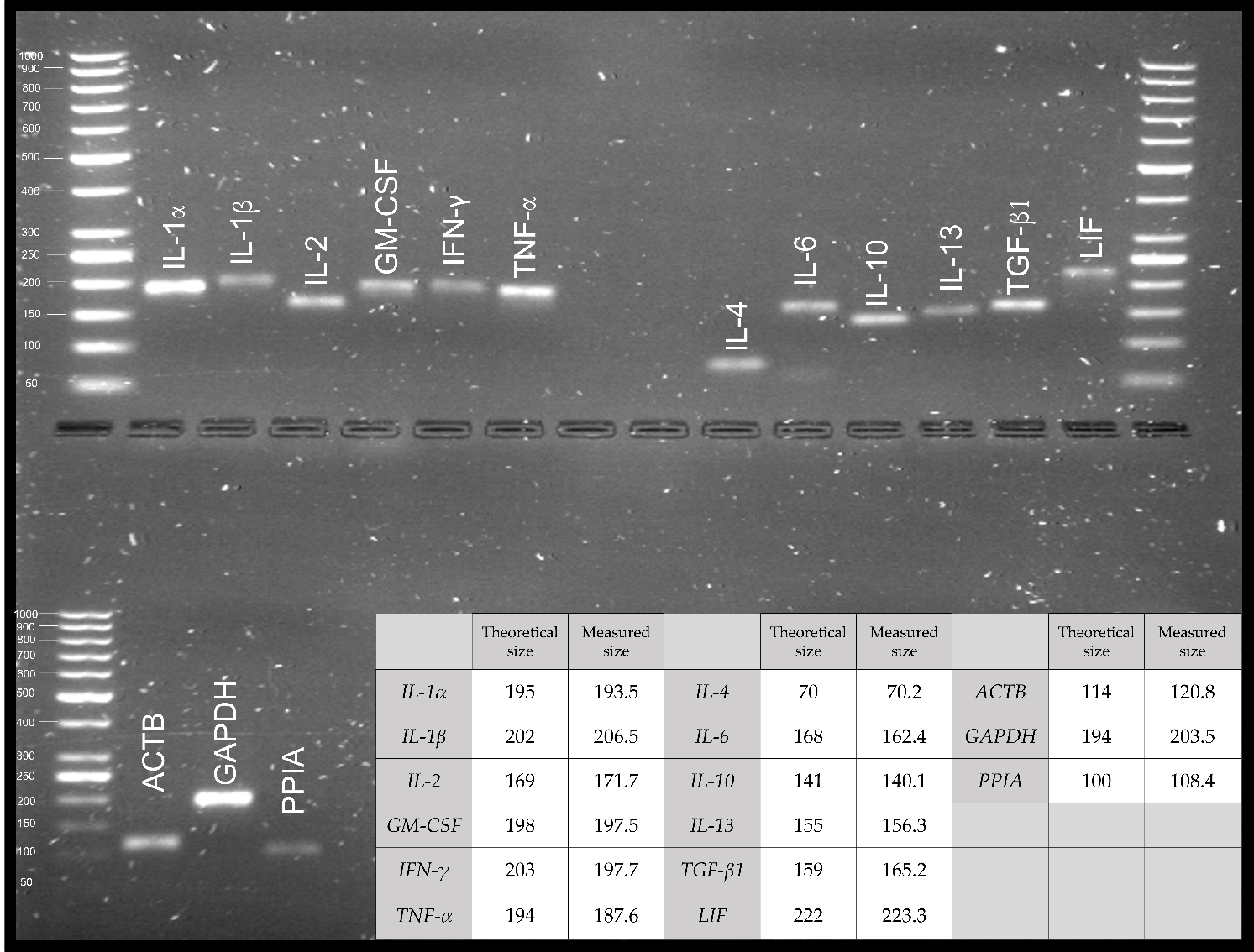


**Supplementary Figure 1.** Agarose gel showing the different amplicons of all analyzed transcripts. Interleukin 1 alpha (IL-1α), interleukin 1 beta (IL-1β), interleukin 2 (IL-2), granulocyte macrophage colony stimulating factor (GM-CSF), interferon gamma (IFN-γ), tumoral necrosis factor alpha (TNF-α), interleukin 4 (IL-4), interleukin 6 (IL-6), interleukin 10 (IL-10), interleukin 13 (IL-13), transforming growth factor beta 1 (TGF-β1), leukemia inhibitory factor (LIF), beta-actin (ACTB), glyceraldehyde-3-phosphate dehydrogenase (GAPDH) and peptidylprolyl isomerase A (PPIA). The theoretical size was calculated in silico using the NCBI database tools. The measured size on the gel was calculated using Image Lab software.


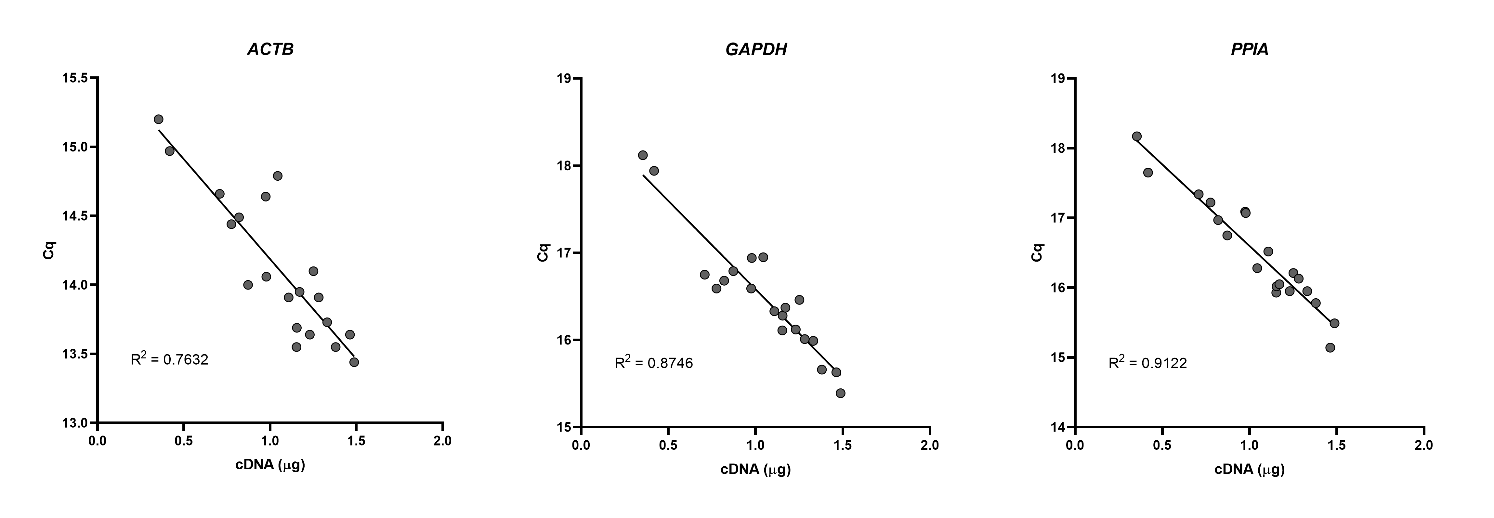


**Supplementary Figure 2.** Correlation analysis between the Cq values of the different endogenous genes and the amount of cDNA in the analyzed samples.
